# Antidiabetes constituents, cycloartenol and 24-methylenecycloartanol, from *Ficus krishnae*

**DOI:** 10.1371/journal.pone.0235221

**Published:** 2020-06-25

**Authors:** Ajikumaran Nair Sadasivan Nair, Reshma Vijayakumari Raveendran Nair, Aroma Prasanna Rajendran Nair, Akhila Sasikumar Nair, Sabu Thyagarajan, Anil John Johnson, Sabulal Baby

**Affiliations:** 1 Phytochemistry and Phytopharmacology Division, Jawaharlal Nehru Tropical Botanic Garden and Research Institute, Thiruvananthapuram, Kerala, India; 2 Garden Management Division, Jawaharlal Nehru Tropical Botanic Garden and Research Institute, Thiruvananthapuram, Kerala, India; University of Colorado Denver School of Medicine, UNITED STATES

## Abstract

*Ficus krishnae* stem bark and leaves are used for diabetes treatment in traditional medicines. Stem bark of *F*. *krishnae* was sequentially extracted with hexane, methanol and water, and these extracts were tested for their antihyperglyceamic activity by oral glucose tolerance test (OGTT) in overnight fasted glucose loaded normal rats. Hexane extract showed significant glucose lowering activity in OGTT, and the triterpene alcohols (cycloartenol+24-methylenecycloartanol) (CA+24-MCA) were isolated together from it by activity guided isolation and characterized by NMR and mass spectroscopy. The ratio of the chemical constituents CA and 24-MCA in (CA+24-MCA) was determined as 2.27:1.00 by chemical derivatization and gas chromatographic quantification. (CA+24-MCA) in high fat diet-streptozotocin induced type II diabetic rats showed significant antidiabetes activity at 1 mg/kg and ameliorated derailed blood glucose and other serum biochemical parameters. Cytoprotective activity of (CA+24-MCA) from glucose toxicity was evaluated in cultured RIN-5F cells by MTT assay and fluorescent microscopy. (CA+24-MCA) in *in vitro* studies showed enhanced cell viability in RIN-5F cells and significant protection of beta cells from glucose toxicity. Both in *in vivo* and *in vitro* studies (CA+24-MCA) showed enhancement in insulin release from the beta cells. In short term toxicity studies in mice (CA+24-MCA) did not show any conspicuous toxic symptoms. The combination of the phytosterols (CA+24-MCA) obtained through activity guided isolation of the stem bark of *F*. *krishnae* showed significant activity, and therefore is a promising candidate for new generation antidiabetes drug development.

## Introduction

Diabetes is a group of metabolic diseases characterized by hyperglycemia resulting from defects in insulin secretion, insulin action or both. The chronic hyperglycemia of diabetes is associated with long term damage, dysfunction and failure of different organs, especially the eyes, kidneys, nerves, heart and blood vessels [1]. The prevalence of Diabetes Mellitus (DM) is in a global boom, and in the past three decades people with DM have increased four times. An estimated 415 million adults were affected by the disease in 2015 and the count jumped to 425 million in 2017 [2]. In other words, about 1 in 11 adults worldwide have DM now. Among the total population of DM worldwide, 90% is coming under type II DM, of which the Asian diabetes cases contribute a significant percentage. China and India are the two major epicenters of type II DM in Asia, and they ranked I and II globally in the prevalence of DM [3]. It is partially due to the genetic predisposition, unhealthy diet, sedentary lifestyle and changing environment factors. DM can be regulated to a great extent by healthy lifestyle, including proper exercise, maintenance of body weight, avoiding smoking and alcohol [4]. But, even after efforts to restrict the incidences of DM, it is on the raise. In this backdrop, search for new generation of antidiabetes drugs is a priority. Plants offer a rich source of structurally diverse molecular entities [5], and we recently isolated secondary metabolites with promising antidiabetes activities from medicinal plants [6, 7].

*Ficus* is a genus with approx. 800 species around the world, and about 115 species are distributed in India. Various *Ficus* species are ethnobotanically/traditionally known for their antidiabetic, anti-inflammatory, anticancer and other activities [8–10]. *Ficus krishnae* C.DC. (Moraceae) is considered as native to India. Its taxonomic status has been revisited by Tiwari and co-workers recently [11]. It has been used in number of folklore medicines. Various parts of the plant are used to treat ulcers, vomiting, fever, inflammation, leprosy, piles, gonorrhea, and as aphrodisiac and tonic. Its aerial roots are styptic, useful in syphilis, biliousness, dysentery and inflammation of liver [12, 13]. *F*. *krishnae* is also used for diabetes in traditional medicines [12, 14, 15]. Preliminary studies showed the antihyperglycemic activity of the petroleum ether extract of *F*. *krishnae* leaves on alloxan-induced diabetic rats [12, 14]. This study is aimed at finding the antidiabetes constituents in *F*. *krishnae* and their mechanism of action.

## Materials and methods

### Chemicals and reagents

Streptozotocin, acridine orange, ethydium bromide, 3-(4,5-dimethylthiazol-2-yl)-2,5-diphenyl tetrazolium bromide (MTT), glibenclamide, streptomycin and penicillin were procured from Sigma Aldrich, St. Louis, USA; Whatman no. 1 filter paper from GE Health Care Life Sciences, UK; silica gel (60–120 & 100–200) from SDFCL, Mumbai, India; pyridine, dimethyl sulphoxide and TLC plates from Merck, Germany. *N*,*O*-bis(trimethylsilyl) trifluoroacetamide was obtained from SRL, Mumbai, India; Tween-80 from Loba Chemicals, India; Krebs-Ringer bicarbonate (KRB) buffer, HEPES from HiMedia, Mumbai, India; cell culture plates from Eppendorf, Germany. RPMI medium, fetal calf serum and trypsin-EDTA were procured from Gibco, USA. All other chemicals and solvents used were of analytical grade.

### Plant material

*F*. *krishnae* stem bark was collected from the conservatory of Jawaharlal Nehru Tropical Botanic Garden and Research Institute (JNTBGRI), Palode, India, identified by Dr. Sabu Thyagarajan (one of the authors), and a voucher specimen (TBGT 72684) was deposited at the Institute Herbarium.

### Extraction

Stem bark of *F*. *krishnae* was shade dried and powdered (0.5 mm size) in a cross beater mill (Nr74364, Retsch, Germany). Stem bark powder (500 g) was extracted sequentially with hexane and methanol (3 L, 24 h each) in Soxhlet apparatus and extracts were filtered through Whatman no. 1 filter paper. Residual stem bark powder was dried solvent free and extracted with water (2 L) for 24 h on a temperature controlled magnetic stirrer (C-MAG-HS7, IKA, Germany) (300 rpm, 35°C) and the extract was filtered through Whatman no. 1 filter paper. Hexane and methanol extracts were concentrated using a rotary evaporator (R-210, Buchi, Switzerland) at 40°C under reduced pressure and the water extract was lyophilized (DPG001, Lyolab, India), to yield 12.0 g (2.4%, dr. wt., stem bark powder) hexane (FKEH), 59.0 g (11.8%, dr. wt.) methanol (FKEM) and 24.0 g (4.8%, dr. wt.) (FKEW) water extracts, respectively (S1 Scheme). These extracts were used for oral glucose tolerance test in overnight fasted glucose loaded normal rats.

### Fractionation

In oral glucose tolerance test, the hexane extract (FKEH) (100 mg/kg) showed significant blood glucose lowering activity and hence, FKEH was subjected to fractionation with column chromatography. Column was loaded with 360 g silica gel (60–120 mesh size) in hexane, FKEH (12 g) was pre-adsorbed in 36 g silica gel (60–120) and loaded onto the column. Seven fractions (FKEHF1 to FKEHF7) were collected using 100% hexane, 95%, 90%, 80%, 60%, 20% hexane in ethyl acetate and 100% ethyl acetate (2 L each). FKEHF1 to FKEHF7 were dried solvent free using a rotary evaporator at 40°C under reduced pressure. Yields of seven fractions (FKEHF1 to FKEHF7) obtained from FKEH were 0.17 g (1.41%, FKEHF1), 5.42 g (45.16%, FKEHF2), 2.04 g (17.00%, FKEHF3), 2.83 g (23.60%, FKEHF4), 0.79 g (6.60%, FKEHF5), 0.36 g (3.00%, FKEHF6) and 0.09 g (0.74%, FKEHF7), respectively (S1 Scheme). All these fractions were tested for their glucose lowering activity by oral glucose tolerance test in overnight fasted glucose loaded normal rats.

### Isolation of active compounds

FKEHF4 showed most significant glucose lowering activity (at 25 mg/kg body weight) in oral glucose tolerance test. In TLC/HPTLC (CAMAG, Switzerland) in 1.5:8.5 hexane-ethyl acetate, FKEHF4 showed a major compound and a minor compound, and it was subjected to column chromatography for further purification. Column was packed with 60 g silica gel (100–200 mesh size) in hexane, FKEHF4 (2 g) was adsorbed in 6 g silica gel (100–200), loaded over the packed column and eluted with hexane and ethyl acetate (100:0 to 75:25) into six fractions (100 ml each). First three fractions (100% hexane, 95% and 90% hexane in ethyl acetate) were pooled together based on their TLC profiles, to yield only the minor compound. Last three fractions (85%, 80% and 75% hexane in ethyl acetate) were pooled together (based on their TLC profiles) to yield the major compound. Pooled fractions were concentrated on a rotary evaporator at 40°C under reduced pressure. Yields of minor and major compounds were 0.40 g (20%) and 1.60 g (80%), respectively (S1 Scheme). Both minor and major compounds were tested for their glucose lowering activity by oral glucose tolerance test in overnight fasted glucose loaded normal rats, and the major compound showed significant antihyperglycaemic activity.

### Identification of active compounds

The major compound (a mixture of two compounds, CA+24-MCA) was characterized by 1H NMR (400 MHz), 13C NMR (100 MHz) (Bruker, Germany) and mass spectrometry (Thermo Fisher Scientific, USA).

### Derivatization, ratio of CA and 24-MCA in (CA+24-MCA)

(CA+24-MCA) (2 mg) was derivatized to trimethylsilyl ethers by adding 300 μL *N*,*O*-bis(trimethylsilyl) trifluoroacetamide and 50 μL pyridine at 60°C for 30 min. Cycloartenol (CA) standard (2 mg) was also derivatized using the same protocol. TLC/HPTLC profiling showed complete conversion of (CA+24-MCA) and standard CA into their trimethylsilyl derivatives. Derivatives of (CA+24-MCA) and standard CA were dissolved in 1.5 mL chloroform and 1 μl each were injected (splitless) on a Gas Chromatograph with AOC-20i autoinjector and FID (GC-2010 Plus, Shimadzu, Japan), fitted with a Rxi-5 Sil MS capillary column (5% phenyl 95% dimethyl polysiloxane, non-polar, 30 m x 0.25 mm i.d., 0.25 μm film thickness (Restek, USA). GC operation conditions: injector temperature 270°C; GC oven was initially maintained at 50°C (2 min), oven temperature programme: 50–280°C (10°C/min), hold time at 280°C (30 min) and FID was set at 280°C. Carrier gas nitrogen, flow rate 1.53 mL/min. GC analyses of samples were repeated four times each and relative percentages of individual components (derivatives of CA, 24-MCA) were calculated from the peak area-percent report of the GC-FID data.

### Animals, cell lines

Male Wistar rats (180–200 g body weight) and male Swiss albino mice (25–28 g body weight) were used for experiments. Animals were fed with standard pellet diet and water *ad libitum*. All animals were maintained under standard laboratory conditions in a room maintained 12 h light/dark cycle, with temperature range of 23–25°C and relative humidity of 45–55%, throughout the experimental period. Animal experiments were approved by the Institute Animal Ethics Committee (IAEC B1/04/2016/PPD-02), which is governed by the Committee for the Purpose of Control and Supervision of Experiments on Animals (CPCSEA), Government of India. Rat insulinoma cell (RIN-5F cells) lines of rat (*Rattus norvegicus*) pancreatic islets origin were procured from the National Centre for Cell Sciences (NCCS), Pune, India. These beta cells are grown as adherent monolayer, and they have the capacity to secrete insulin. RIN-5F cells cultured in RPMI medium supplemented with 10% fetal calf serum, 100 μg/ml streptomycin and 100 units/ml penicillin in a carbon dioxide incubator (Eppendorf, Galaxy 170R, Germany) were used for *in vitro* studies.

### Oral glucose tolerance test

Oral glucose tolerance test was performed as described elsewhere [16]. Briefly, required number of overnight fasted normal Wistar rats were selected and segregated into required numbers of groups containing six animals each. Animals in all groups received vehicle (1% Tween-80) or test material (extracts, FKEH, FKEM and FKEW: 100 mg/kg each; fractions, FKEHF1: 5 mg/kg, FKEHF2: 30 mg/kg, FKEHF3: 20 mg/kg, FKEHF4: 25 mg/kg, FKEHF5: 25 mg/kg, FKEHF6: 5 mg/kg, FKEHF7: 5 mg/kg; major compound 20 mg/kg, minor compound 5 mg/kg; various doses of (CA+24-MCA) 250 μg/kg, 1 mg/kg and 5 mg/kg) per orally (dissolved in 1% Tween-80, 1 ml/animal). Glucose (3 g/kg) was given to all animals per orally (1 ml/animal) after 30 min of administration of vehicle/test materials. Blood samples were collected from tail vein prior to the administration of vehicle/test materials at 0 min, and at 30, 90 and 150 min after glucose administration, and blood glucose levels were determined with a glucometer (One Touch Ultra Glucometer, Horizon, USA).

### High fat diet fed-streptozotocin (HFD-STZ) induced type II diabetic rats

Male Wistar rats of 175–200 g body weight were selected and fed with high fat diet (HFD, 24% fat, 24% protein, 41% carbohydrate and 11% normal feed powder). After two weeks of HFD diet, animals were injected intraperitoneally with a single dose (35 mg/kg) of streptozotocin (STZ) in citrate buffer (500 μl/animal). Seven days after STZ injection blood samples were collected from the tail vein, blood glucose levels were determined using a glucometer and serum insulin levels were determined by ultrasensitive Rat Insulin ELISA assay kit (80-INSRTU-E01, ALPCO Diagnostics Inc., USA) [17].

### Antidiabetes activity of (CA+24-MCA) in HFD-STZ type II diabetic rats

HFD-STZ induced type II diabetic rats (eighteen) with average glucose value of 270–286 mg/dl and serum insulin values of 0.30–0.32 ng/ml were selected and grouped into three groups of six animals each. One group was kept as diabetic control (received 1% tween-80), and animals in test group received 1 mg/kg (CA+24-MCA) (dose was determined by the screening test), and the last group of diabetic animals received 500 μg/kg of glibenclamide. Along with the diabetic animals, two groups of age and sex matched male Wistar rats were selected, of which one group of six animals fed with normal pellet diet with average blood glucose value of 86.5 ± 3.2 mg/dl and serum insulin values of 0.22 ± 0.02 ng/ml was kept as normal control animals (received 1% tween-80) and other group of six high fat diet (HFD) fed animals with average blood glucose value of 121.3 ± 4.1 mg/dl and serum insulin values of 0.28 ± 0.04 ng/ml were kept as HFD control animals (received 1% tween-80). HFD control and HFD-STZ diabetic animals were fed with HFD and normal control animals were fed with normal pellet diet for entire experimental period. All animals received a daily single dose (1 ml/animal) of vehicle or drug or standard per orally for 25 days. Blood samples were collected from tail vein of all animals on 1, 5, 10, 15, 20 and 25th day and blood glucose levels were determined using a glucometer. On 25th day after blood glucose determination, all animals were sacrificed, blood serum and liver samples were collected and serum biochemical parameters and liver glycogen levels were estimated.

### Cytoprotective effect of (CA+24-MCA) from gluco-toxicity in RIN-5F cells (MTT assay)

RIN-5F cells were cultured in RPMI media supplemented with 10% FCS, 100 μg/ml streptomycin and 100 units/ml penicillin in a carbon dioxide incubator at 37°C, 5% carbon dioxide and 95% air for 48 h. The cultured cells were trypsinised and adjusted the cell number to 1x10^**5**^ cells/ml with complete media containing normal glucose (5.5 mM) and other set of cells in complete media with high glucose (25 mM). Gluco-toxicity on RIN-5F cells was determined by MTT assay. Precisely, cells in both normal and high glucose medium (1x10^**5**^ cells/ml) were cultured in 24 well plates for 24 h in the carbon dioxide incubator. In case of normal glucose medium, the cells in first three wells were kept as control incubated with 0.1% DMSO, the cells in second, third and fourth sets of wells (triplicate) were incubated with 0.1 μg/ml, 1 μg/ml and 10 μg/ml of (CA+24-MCA) for 48 h. After 48 h of incubation the spent medium was replaced with fresh medium containing 20 μl of 5 mg/ml of MTT and incubated for 4 more h in the carbon dioxide incubator. After incubation, the cells were harvested and added with 1 ml of DMSO and optical density was determined at 570 nm in plate reader (iMARK plate reader, Bio-Rad, USA). Based on the optical density the cytoprotective activity (cell proliferation) of (CA+24-MCA) was calculated. In order to determine the cytoprotective activity of (CA+24-MCA) on RIN-5F cells in high glucose medium, similar MTT assay was performed wherein instead of low glucose media, complete media with high glucose was used.

### Cytoprotective effect of (CA+24-MCA) from gluco-toxicity in RIN-5F cells (fluorescent microscopy)

In order to determine the cytoprotective effect of (CA+24-MCA) in RIN-5F cells cultured in normal and high glucose media, 1x10^**5**^ cells/ml were cultured in RPMI media containing 10% FCS, 100 μg/ml streptomycin and 100 units/ml penicillin with normal (5.5 mM) or high glucose (25 mM) for 24 h. RIN-5F cells in normal or high glucose medium were incubated with 0.1% DMSO, 0.1 μg/ml, 1 μg/ml or 10 μg/ml of (CA+24-MCA) for 48 h at 37°C, 5% carbon dioxide and 95% air. After 48 h of incubation, spent medium was removed, cells were stained with 50 μl of acridine orange-ethydium bromide (AO-EtBr) stain (100 μg/ml) and observed under an inverted microscope (CKX41, Olympus, USA) with blue filter and photographed with a Zoom digital compact camera (CAMEDIA C-4000, Olympus, USA). Viable live cells and dead non-viable cells appeared in green and orange-red colour, respectively.

### Insulin release from RIN-5F cells by (CA+24-MCA)

Insulin release from RIN-5F cells was determined as described in Ajikumaran et al., 2014 [6]. Briefly, RIN-5F cells (1x10^**5**^ cells/ml) were cultured in 24 well plates with RPMI medium containing 10% FCS, 100 μg/ml streptomycin and 100 units/ml penicillin with normal (5.5 mM) or high glucose (25 mM) for 48 h at 37°C and 5% CO_**2**_. Control and tests were done in triplicate. After incubation, spent medium was removed and cells were washed thrice with 1 ml of glucose free Krebs-Ringer bicarbonate (KRB) buffer, pH 7.4. These cells were incubated with 1 ml of glucose free KRB buffer supplemented with 1 mg/ml BSA, 10 mM HEPES for 2 h. After incubation the medium was changed and incubated with 1 ml of fresh glucose free KRB medium with 0.1% DMSO, or 0.1 μg/ml or 1 μg/ml or 10 μg/ml of (CA+24-MCA) for 1 h in CO_**2**_ incubator at 37°C. The supernatants were separated and insulin contents were determined with ultrasensitive Rat Insulin ELISA assay kit following the manufacture’s instruction (80-INSRTU-E01, ALPCO Diagnostics Inc., USA) using a plate reader (iMARK Plate Reader, Bio-Rad, USA).

### Subacute toxicity of (CA+24-MCA) in mice

Subacute toxicity evaluation of (CA+24-MCA) was carried as described in Shylesh et al., 2005 [18]. Briefly, twenty four male Swiss Albino mice (25–28 g body weight) were divided into four groups of six animals each. One group was kept as control received 1% tween-80 and second, third and fourth group of animals received 15 mg/kg, 75 mg/kg or 150 mg/kg of (CA+24-MCA), respectively, for 30 days (1 ml daily per orally). Food and water intake and behavior of animals were observed daily for 1 h for 29 days. Animals were sacrificed on the 30th day, important organs were dissected out and observed for pathological and morphological changes, blood samples were collected to determine the hematological and serum biochemical parameters.

### Hematological and serum biochemical parameters, liver glycogen

Hemoglobin was measured using hemoglobinometer with comparison standards. Glutamate pyruvate transaminase (GPT), glutamate oxaloacetate transaminase (GOT) and alkaline phosphatase (AP) were measured by standard methods [19]. Urea, cholesterol, triglycerides, creatinine and protein were determined by conventional methods [20]. Peritoneal macrophages and total leucocytes were collected and counted as described by Babu et al., 2003 [21]. Serum glucose was estimated spectrophotometrically using a commercial assay kit (Monozyme, India, Ltd). Liver glycogen was estimated by the method of Carrol et al., 1956 [22]. Serum insulin was determined with ultrasensitive rat insulin ELISA assay kit (80-INSRTU-E01, ALPCO Diagnostics Inc., USA).

### Statistical analysis

Statistical comparisons were done using one-way ANOVA followed by Dunnett’s post hoc comparisons and student’s *t*-test was performed in paired data analysis. P values < 0.05 were considered to be significant.

## Results

### Isolation, characterization of (cycloartenol+24-methylenecycloartanol)

**Fig 1:**
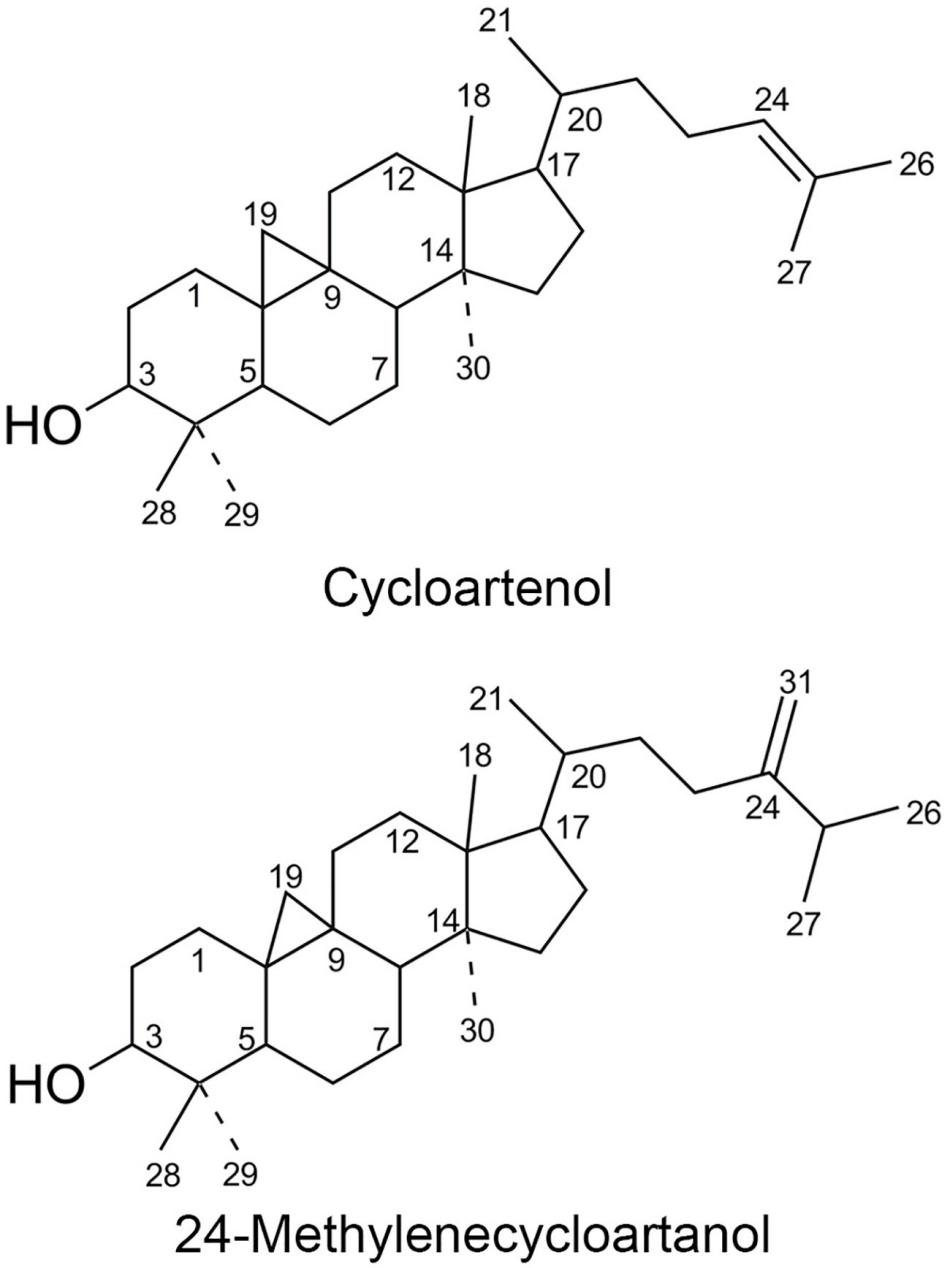
Cycloartenol (CA) and 24-Methylenecycloartanol (24-MCA).

Cycloartenol (CA) and 24-methylenecycloartanol (24-MCA) were isolated together from the hexane extract of the stem bark of *F*. *krishnae* by activity guided isolation and characterized by NMR and mass spectroscopy (S1 Scheme, Fig 1, S1 Table, S1, S2 and S3 Figs).

White crystalline solid, purity 100%, yield 100 mg (0.0031% w/w, dr. wt.), gave positive result for terpenoids in Liebermann-Burchard test [23]. TLC (hexane:ethyl acetate, 9.5:0.5) showed a bright pink coloured spot (Rf = 0.5) on derivatization with anisaldehyde-sulphuric acid reagent and heating at 110°C. 13C NMR showed a total of 37 peaks, with several intense signals, indicating a possible mixture of two similar terpenoids (CA & 24-MCA). 13C NMR spectrum of CA displayed 30 carbons corresponding to seven methyl carbons at δ 18.05 (C-18), 18.31 (C-21), 17.67 (C-26), 25.75 (C-27), 26.03 (C-28), 14.00 (C-29), 19.64 (C-30), eleven methylene carbons at δ 32.90 (C-1), 31.32 (C-2), 21.89 (C-6), 26.03 (C-7), 26.48 (C-11), 33.81 (C-12), 35.89 (C-15), 28.17 (C-16), 29.92 (C-19), 36.36 (C-22), 25.42 (C-23), five methine carbons at δ 78.86 (C-3), 47.71 (C-5), 48.01 (C-8), 52.29 (C-17), 36.13 (C-20), five quaternary carbons at δ 40.49 (C-4), 20.00 (C-9), 26.48 (C-10), 45.30 (C-13), 48.81 (C-14) and two olefinic carbons at 125.27 (C-24), 130.69 (C-25). 13C NMR spectrum of 24-MCA showed 31 carbons. 13C NMR values of 24-MCA are similar to CA up to the 21st carbon (Fig 1, S1 Table). 24-MCA has an additional olefinic bond attached to the 24th carbon which corresponds to peaks at δ 105.93 (C-31) and 156.91 (C-24). In both compounds (CA & 24-MCA), the C-19 carbon forms a cyclopropyl ring with C-9 and C-10 carbons. 1H NMR of CA showed olefinic proton at δ 5.10 (1H, t, J = 5.6 Hz) and 24-MCA showed olefinic protons at 4.67 (1H, brs, H-a) and 4.71 (1H, d, J = 1 Hz, H-b). Both (CA & 24-MCA) showed signals at δ 3.29 (1H, m) indicating a -CH- group (C-3) attached to an -OH, cyclopropyl methylene protons at 0.33 (1H, d, 3.2 Hz, Ha) and 0.55 (1H, d, 3.2 Hz, Hb) and a characteristic doublet at δ 0.89 (3H, d, J = 6.2 Hz, CH_**3**_). Two methyl groups (H-26 and H-27) attached to C-25 are split into two doublets at δ 1.04 (3H, d, J = 2.3 Hz, CH_**3**_) and 1.02 (3H, d, J = 2.3 Hz, CH_**3**_) in 24-MCA. 1H NMR spectrum of CA showed six tertiary methyl groups at δ 0.81 (H-18), 0.87 (H-30), 0.88 (H-29), 0.97 (H-28), 1.61 (H-26), 1.69 (H-27) and 24-MCA showed four tertiary methyl groups at 0.81 (H-18), 0.87 (H-30), 0.89 (H-29) and 0.97 (H-28). ESI-MS of (CA+24-MCA) showed [M+H]^**+**^ ion peaks at m/z 427.3952 and m/z 441.4109, corresponding to cycloartenol (CA) (calc. mass 426.3862) and 24-methylenecycloartanol (24-MCA) (calc. mass 440.4018) (S3 Fig). Based on these spectral data and comparison with literature, the compound(s) were identified as a mixture of cycloartenol (CA) and 24-methylenecycloartanol (24-MCA) (4,4-dimethyl phytosterols, triterpene alcohols) [24–28]. The ratio of CA and 24-MCA in (CA+24-MCA) based on GC quantification of trimethylsilyl derivatives was (69.46 ± 0.09:30.54 ± 0.09) (n = 4) or (2.27 CA to 1.00 24-MCA) (S4 Fig).

### Oral glucose tolerance test

**Fig 2:**
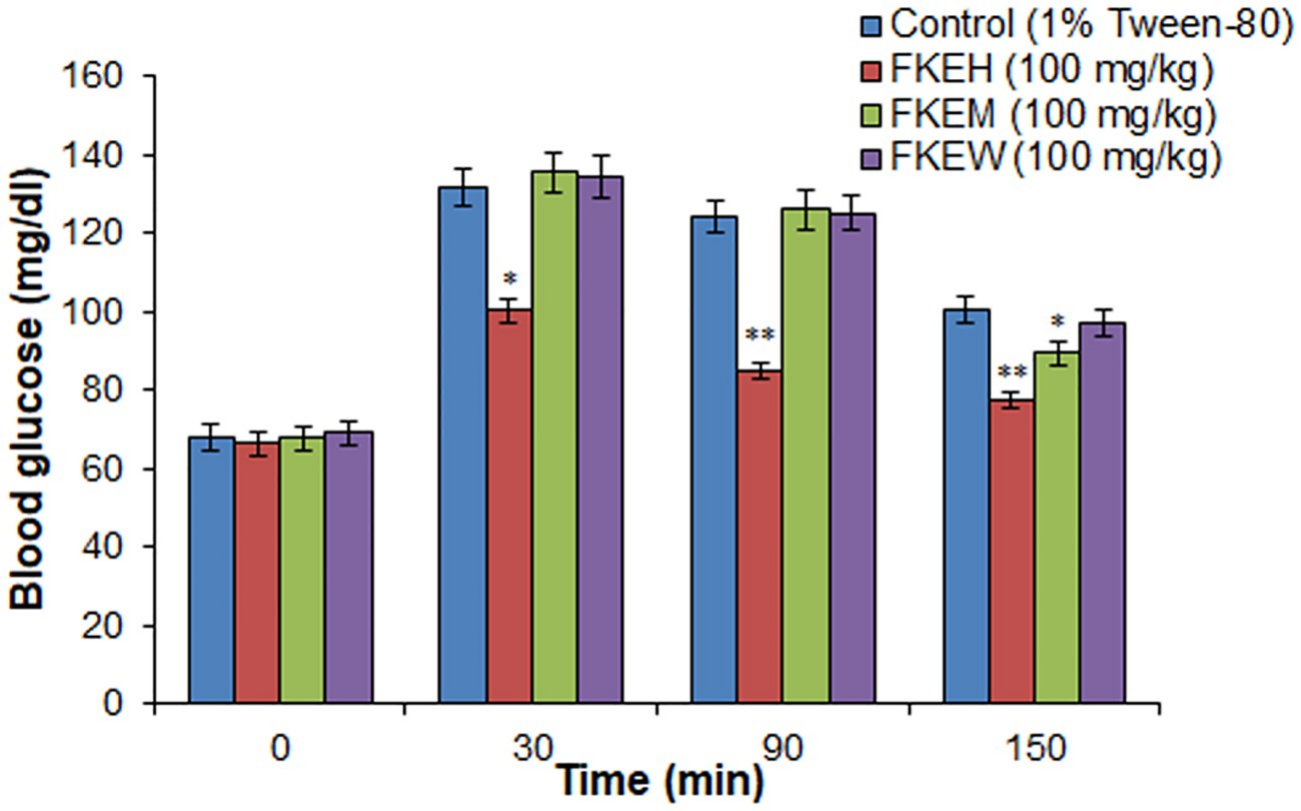
Effect of different extracts of *F*. *krishnae* on glucose tolerance in fasted and glucose loaded normal rats. FKEH: hexane extract, FKEM: methanol extract, FKEW: water extract; Values are mean ± S.D.; n = 6; * P < 0.05; ** P < 0.001 (compared to control). Blood glucose values were determined prior to extract administration (0 min), glucose (3 g/kg) was loaded 30 min after the extract administration, and blood glucose levels were determined at 30, 90 and 150 min (after glucose administration).

Oral glucose tolerance tests of various extracts in overnight fasted glucose loaded normal rats (Fig 2) showed significant glucose lowering activity by the hexane extract (FKEH) (100 mg/kg) at 30, 90, 150 min after glucose administration, and the respective test/control values are (100.2 ± 3.2, 85.0 ± 2.1, 77.6 ± 2.2 mg/dl) and (131.6 ± 4.8, 124 ± 4.1, 100.6 ± 3.2 mg/dl). Methanol (FKEM) and water (FKEW) extracts didn’t show any significant glucose lowering activity in the doses tested, except a glucose lowering activity observed at 150 min after glucose administration in FKEM (100 mg/kg), wherein the values of test and control animals were 89.5 ± 3.1 mg/dl and 100.6 ± 3.2 mg/dl, respectively (Fig 2).

**Fig 3:**
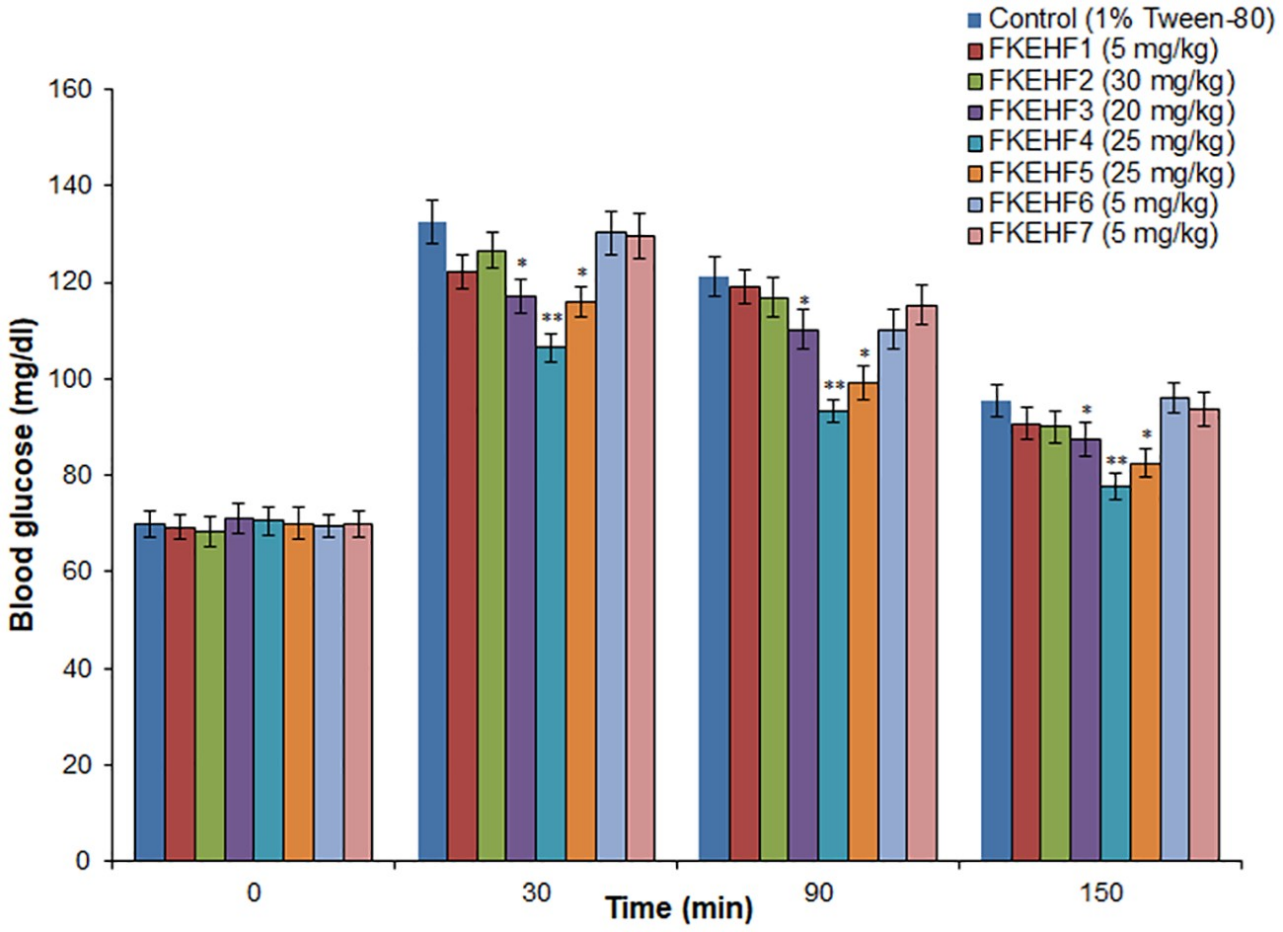
Effect of different fractions of hexane extract of *F*. *krishnae* on glucose tolerance in fasted and glucose loaded normal rats. FKEHF1, 100% hexane fraction; FKEHF2, 95% hexane + 5% ethyl acetate fraction; FKEHF3, 90% hexane + 10% ethyl acetate fraction; FKEHF4, 80% hexane + 20% ethyl acetate fraction; FKEHF5, 60% hexane + 40% ethyl acetate fraction; FKEHF6, 20% hexane + 80% ethyl acetate fraction; FKEHF7, 100% ethyl acetate fraction; values are mean ± S.D.; n = 6; * P < 0.05; ** P < 0.001 (compared to control). The concentrations of various fractions tested were fixed based on their yield from the hexane extract (FKEH). Blood glucose values were determined prior to administration of fractions (0 min), glucose (3 g/kg) was loaded 30 min after administration of fractions, and blood glucose levels were determined at 30, 90 and 150 min (after glucose administration).

Among the seven fractions of FKEH (FKEHF1 to FKEHF7), FKEHF1, FKEHF6 and FKEHF7 with yields < 5% were subjected to oral glucose tolerance tests at a higher dose of 5 mg/kg, and the major fractions (FKEHF2, FKEHF3, FKEHF4, FKEHF5) with yields > 5% were tested in the range of 20–30 mg/kg. FKEHF4 at 25 mg/kg dose showed most significant glucose lowering activity at 30, 90, 150 min after glucose administration, and the values were 106.5 ± 2.9, 93.3 ± 2.5, 77.7 ± 2.6 mg/dl, respectively, compared to the control values of 132.5 ± 4.5, 121.2 ± 4.2, 95.6 ± 3.2 mg/dl, respectively (Fig 3). On chemical profiling of fractions (FKEHF1 to FKEHF7) by TLC/HPTLC, the active component (CA+24-MCA) was found only in FKEHF4, and its overlap in other fractions was not observed. FKEHF3 and FKEHF5 showed less significant glucose lowering activity compared to the control values, whereas FKEHF1, FKEHF2, FKEHF6, and FKEHF7 didn’t show any significant glucose lowering activity in the glucose tolerant test.

**Fig 4:**
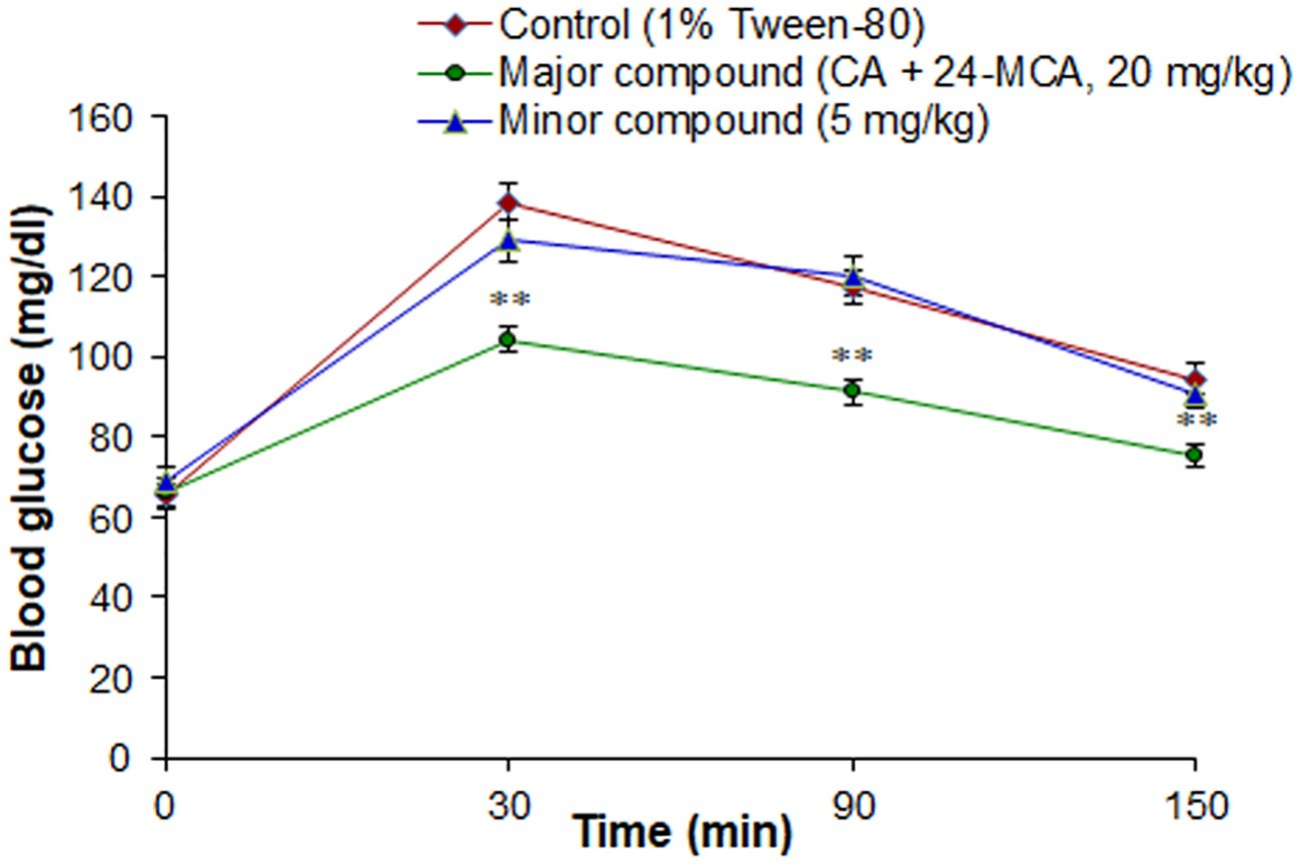
Effect of major and minor compounds isolated from the active fraction (FKEHF4) of hexane extract from *F*. *krishnae* on glucose tolerance in fasted and glucose loaded normal rats. (CA+24-MCA), cycloartenol (CA) + 24-methylenecycloartanol (24-MCA); values are mean ± S.D.; n = 6; ** P < 0.001 (compared to control). Blood glucose values were determined prior to the administration of major and minor compounds (0 min), glucose (3 g/kg) was loaded 30 min after the administration of major and minor compounds, and blood glucose levels were determined at 30, 90 and 150 min (after glucose administration).

**Fig 5:**
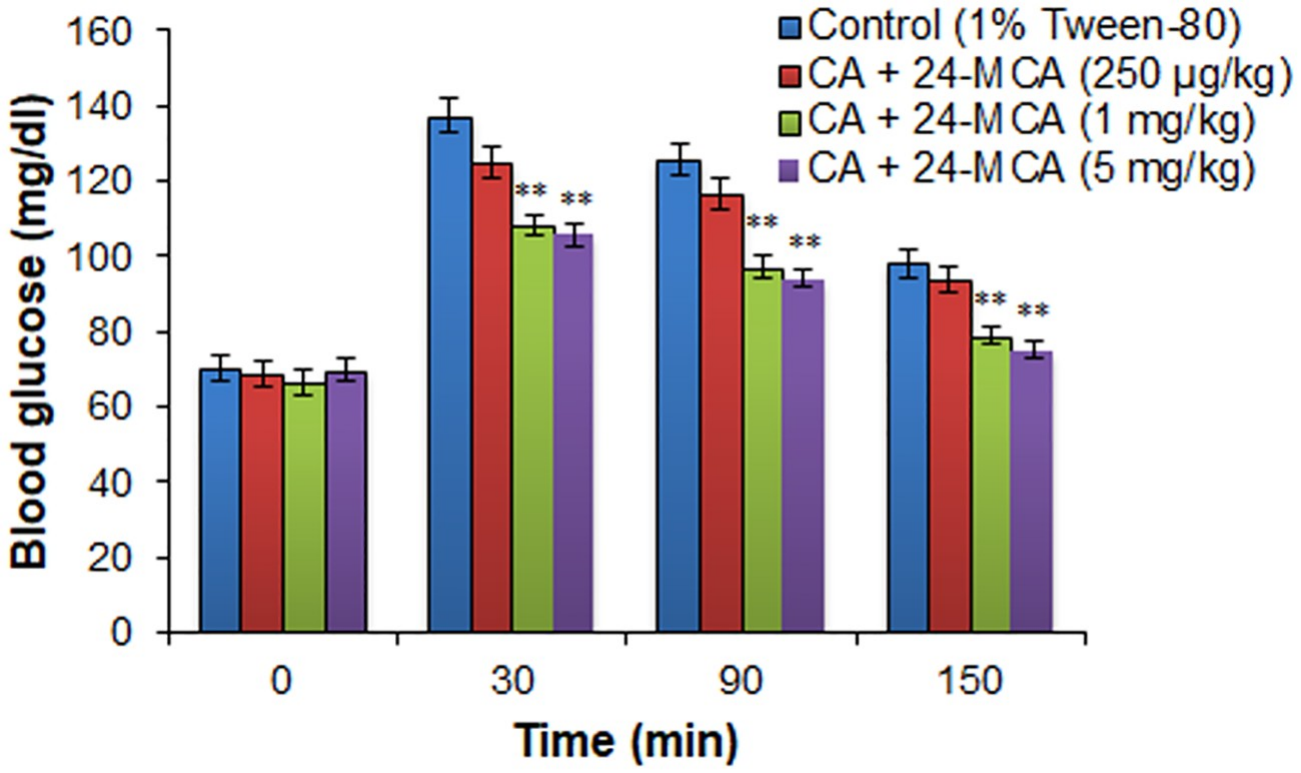
Effect of various doses of (CA+24-MCA) isolated from the active fraction (FKEHF4) of hexane extract from *F*. *krishnae* on glucose tolerance in fasted and glucose loaded normal rats. (CA+24-MCA), cycloartenol (CA) + 24-methylenecycloartanol (24-MCA); values are mean ± S.D.; n = 6; ** P < 0.001 (compared to control). Blood glucose values were determined prior to the administration of (CA+24-MCA) (0 min), glucose (3 g/kg) was loaded 30 min after (CA+24-MCA) administration, and blood glucose levels were determined at 30, 90 and 150 min (after glucose administration).

The major compound of FKEHF4 (CA+24-MCA) at 20 mg/kg dose showed significant glucose lowering activity at 30, 90, 150 min after glucose administration, and the values were 104.3 ± 3.1, 91.2 ± 2.9, 75.4 ± 2.5 mg/dl compared to control values of 138.8 ± 4.5, 117.5 ± 4.1, 94.6 ± 3.6 mg/dl, respectively (Fig 4). The minor compound didn’t show any significant glucose lowering activity at the dose tested. (CA+24-MCA) at 1 mg/kg and 5 mg/kg showed a dose dependent glucose lowering activity (Fig 5), optimum activity was observed at 1 mg/kg body weight and the 250 μg/kg dose didn’t show any significant glucose lowering activity.

### Antidiabetes activity of (CA+24-MCA) in HFD-STZ type II diabetic rats

**Fig 6:**
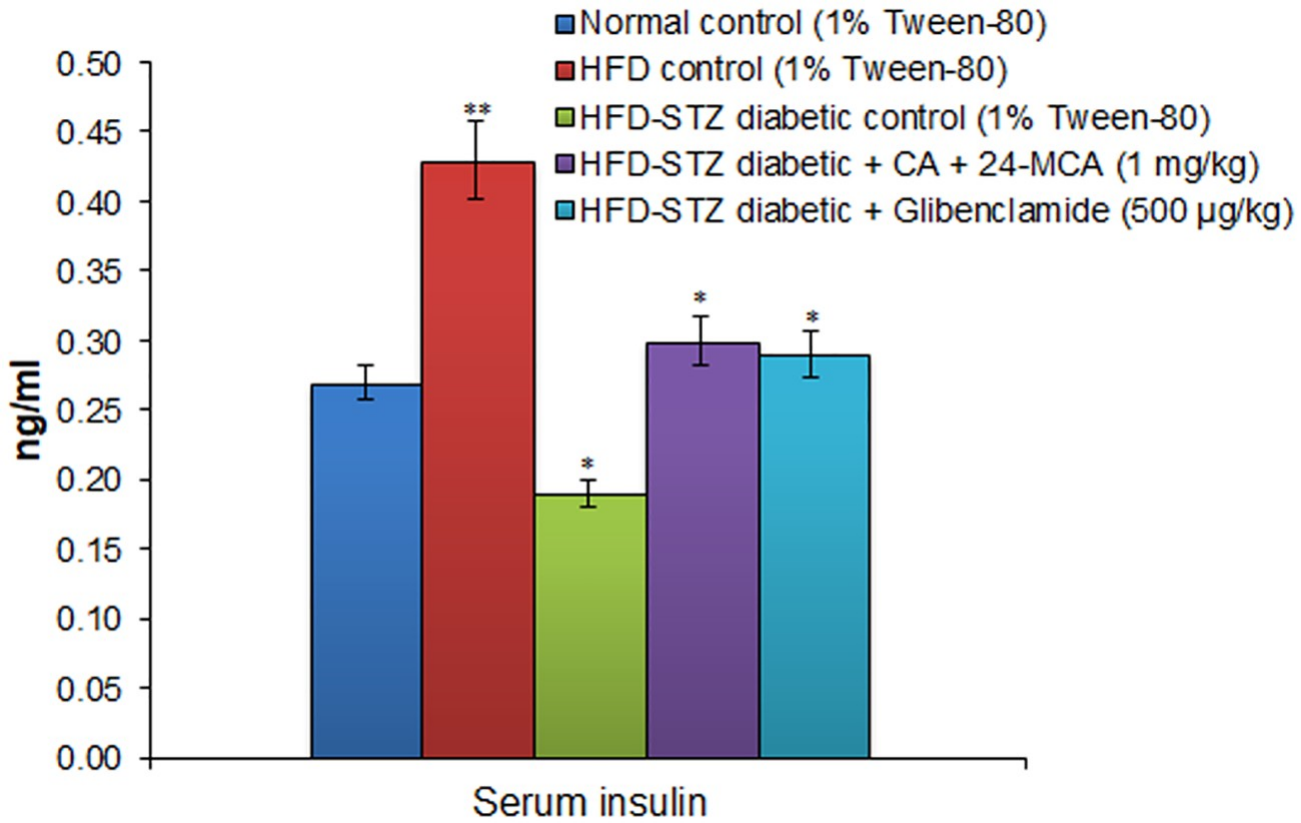
Effect of (CA+24-MCA) isolated from hexane extract of *F*. *krishnae* on serum insulin level in high fat diet-streptozotocin type II diabetic rats. (CA+24-MCA), cycloartenol (CA) + 24-methylenecycloartanol (24-MCA); HFD, high fat diet; values are mean ± S.D.; n = 6; * P < 0.05; ** P < 0.001 (compared to normal control); HFD-STZ, high fat diet-streptozotocin.

(CA+24-MCA) at 1 mg/kg in 25 days treatment in HFD-STZ diabetic rats showed significant antidiabetes activity (Table 1), and significantly normalised derailed blood glucose levels from 348.4 ± 6.8 mg/dl (control diabetic rats) to 153.7± 2.5 mg/dl. The reduction in blood glucose levels in glibenclamide treated diabetic animals was comparable to that of the test compound. The drug treatment normalized the deranged liver weight and liver glycogen to normal values, and was comparable to the standard drug glibenclamide (Table 2). Serum biochemical parameters of the experiments are given in the Table 3, (CA+24-MCA) and glibenclamide ameliorated the derailed serum biochemical parameters to normal values. The untreated diabetic control animals showed significant increase in GOT, GPT, urea, triglycerides, total cholesterol, creatinine, albumin and a decrease in protein levels. HFD-STZ diabetic control animals showed an increase in insulin release till the 15th day followed by a decline on 25th day, and their values on 1st, 5th, 10th, 15th, 20th and 25th days were 0.42 ± 0.06, 0.45 ± 0.04, 0.47 ± 0.06, 0.49 ± 0.05, 0.38 ± 0.08 and 0.19 ± 0.1 ng/ml, respectively. Serum insulin levels in experimental animals on 25th day are given in Fig 6. The diabetic animals on treatment with test drug or glibenclamide normalized the deranged insulin levels to normal control values.

**Table 1:** Effect of (CA+24-MCA) isolated from hexane extract of *F*. *krishnae* on blood glucose levels in high fat diet-streptozotocin type II diabetic rats.

Treatments	Serum glucose levels (mg/dl)					
	1st day	5th day	10th day	15th day	20th day	25th day
Normal control (1% Tween-80)	86.5 ± 3.2	82.6 ± 2.8	82.3 ± 3.5	89.0 ± 2.6	87.5 ± 3.1	91.2 ± 3.0
HFD control (1% Tween-80)	121.3 ± 4.1	125.0 ± 3.6	124.0 ± 4.0	128.0 ± 3.1	131.0 ± 3.2	129.0 ± 3.5
HFD-STZ diabetic control (1% Tween-80)	276.2 ± 4.8	295.2 ± 4.9	308.2 ± 5.2	319.8 ± 6.5	327.2 ± 6.1	348.4 ± 6.8
HFD-STZ diabetic + (CA+24-MCA) (1 mg/kg)	281.0 ± 5.1	230.0 ± 4.1*	209.0 ± 3.9**	191.0± 3.5**	177.0 ± 3.1**	153.7 ± 2.5**
HFD-STZ diabetic + Glibenclamide (500 μg/kg)	276.3 ± 4.6	228.5 ± 4.3*	203.2 ± 3.9**	186.8 ± 3.2**	175.5 ± 3.3**	148.5 ± 3.0**

(CA+24-MCA), cycloartenol (CA) + 24-methylenecycloartanol (24-MCA); HFD, high fat diet; values are mean ± S.D.; n = 6;

* P < 0.05;

** P < 0.001 (compared to diabetic control); HFD-STZ, high fat diet-streptozotocin; Blood was collected 1 h after drug administration, except on day 1 (blood was collected just before drug administration).

**Table 2:** Effect of (CA+24-MCA) isolated from hexane extract of *F*. *krishnae* on body weight, liver weight, liver glycogen in high fat diet-streptozotocin type II diabetic rats.

Treatments	Initial body weight (g)	Liver weight (g)	Liver glycogen (mg/g wet tissue)
Normal control (1% Tween-80)	231.5 ± 3.8	8.0 ± 0.8	8.5 ± 0.8
HFD control (1% Tween-80)	237.2 ± 4.3	9.5 ± 1.1	9.4 ± 0.9
HFD-STZ diabetic control (1% Tween-80)	241.2 ± 3.2	6.4 ± 0.5*	6.1± 0.5*
HFD-STZ diabetic + (CA+24-MCA) (1 mg/kg)	235.6 ± 4.3	8.3 ± 0.7	8.3 ± 0.6
HFD-STZ diabetic + Glibenclamide (500 μg/kg)	239.2 ± 4.3	8.6 ± 0.5	8.1 ± 0.7

(CA+24-MCA), cycloartenol (CA) + 24-methylenecycloartanol (24-MCA); HFD, high fat diet; values are mean ± S.D; n = 6;

* P < 0.05 (compared to HFD control); HFD-STZ, high fat diet-streptozotocin.

**Table 3:** Effect of (CA+24-MCA) isolated from hexane extract of *F*. *krishnae* on serum biochemical parameters in high fat-diet streptozotocin type II diabetic rats.

Treatments	GOT (IU/l)	GPT (IU/l)	Urea (mg/dl)	Triglycerides (mg/dl)	Total Cholesterol (mg/dl)	Creatinine (mg/dl)	Albumin (g/dl)	Protein (g/dl)
Normal control (1% Tween-80)	69.5 ± 3.5	41.2 ± 2.4	21.2 ± 2.1	40.8 ± 3.4	78.0 ± 3.4	0.66 ±0.12	3.34 ± 0.31	8.8 ± 1.1
HFD control (1% Tween-80)	75.5 ± 2.7	36.3 ± 2.1	22.3 ± 2.3	68.2 ± 3.8	117.2 ± 5.0	0.95 ± 0.10	3.60 ± 0.28	8.4 ± 0.9
HFD-STZ diabetic control (1% Tween-80)	120.8 ± 5.2**	68.1 ± 2.8**	41.0 ± 3.4**	173.0 ± 7.1**	200.8 ± 7.9**	2.10 ± 0.27**	2.32 ± 0.21*	5.1 ± 0.4*
HFD-STZ diabetic + (CA+24-MCA) (1 mg/kg)	61.5 ± 3.1	39.0 ± 2.3	22.2 ± 2.4	79.1 ± 4.1	80.5 ± 4.3	0.58 ± 0.11	3.30 ± 0.27	8.0 ± 0.5
HFD-STZ diabetic + Glibenclamide (500 μg/kg)	65.4 ± 3.4	39 ± 2.4	22.1 ± 2.1	81.5 ± 4.0	111.4 ± 4.1	1.20 ± 0.13	3.1 ± 0.25	8.1 ± 0.5

(CA+24-MCA), cycloartenol (CA) + 24-methylenecycloartanol (24-MCA); HFD, high fat diet; values are mean ± S.D.; n = 6;

* P < 0.05;

** P < 0.001 (compared to HFD control); HFD-STZ, high fat diet-streptozotocin.

### Cytoprotective effect of (CA+24-MCA) from glucotoxicity in RIN-5F cell by MTT assay

**Fig 7:**
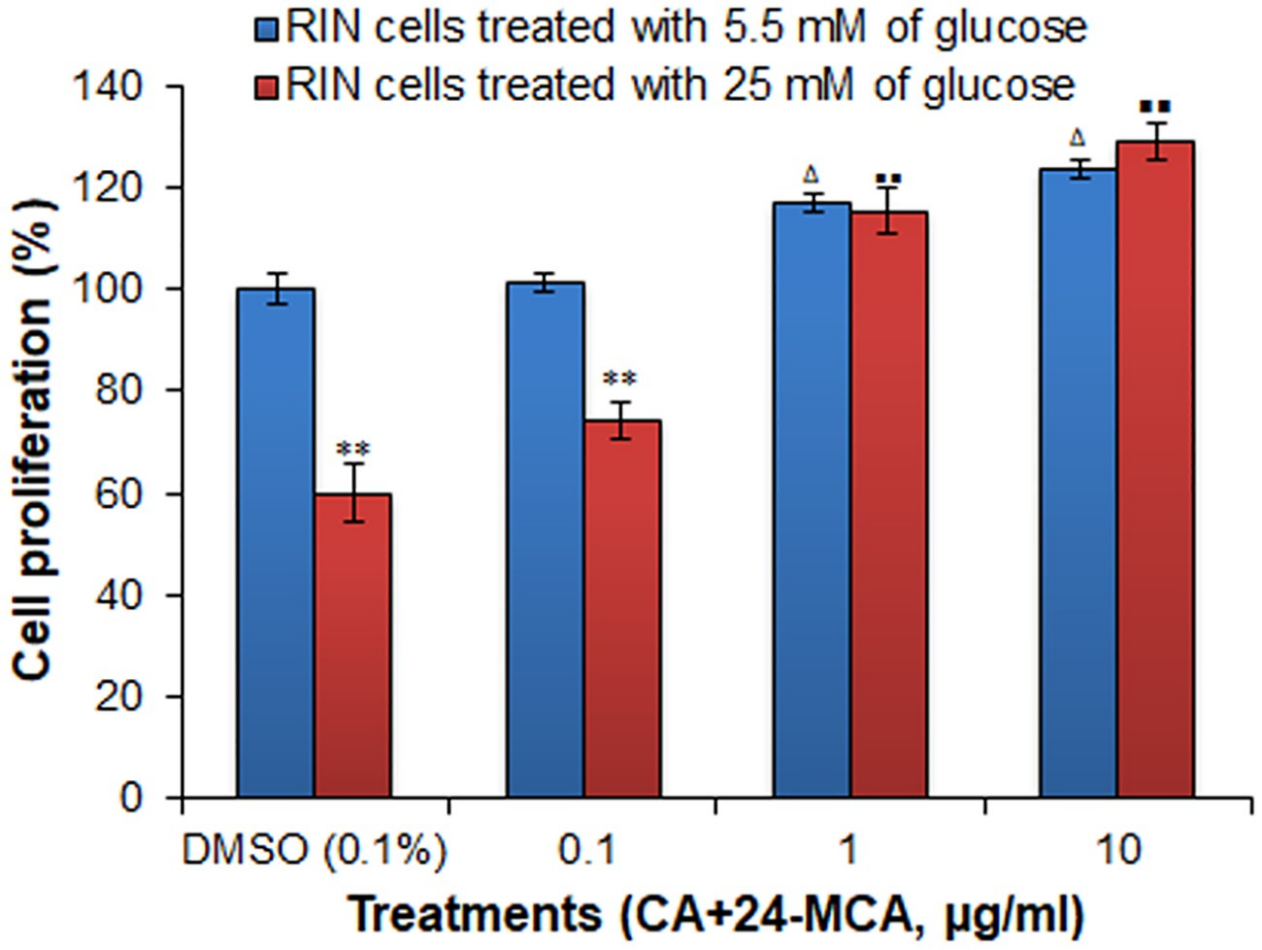
Effect of (CA+24-MCA) isolated from hexane extract of *F*. *krishnae* on RIN-5F cells by MTT assay. (CA+24-MCA), cycloartenol (CA) + 24-methylenecycloartanol (24-MCA); values are mean ± S.D.; n = 3; ** P < 0.001 (compared to the same concentration in 5.5 mM glucose); ^Δ^ P < 0.05 (compared to control cells cultured in 5.5 mM glucose only); ^▪▪^ P < 0.001 (compared to control cells cultured in 25 mM glucose only); cell proliferation in normal glucose (5.5 mM) medium treated with 0.1% DMSO was considered as 100%. Percentage cell proliferation in all other doses, *viz*., (CA+24-MCA) in normal (5.5 mM) and high glucose (25 mM) medium, and control cells (0.1% DMSO) in high glucose (25 mM) medium, were calculated based on their optical densities against those of control cells in normal glucose (5.5 mM) medium.

Cells cultured in normal glucose medium showed increase in cell proliferation dose dependently and percentage increment in cell growth at 0.1 μg/ml, 1 μg/ml, 10 μg/ml doses of (CA+24-MCA) were 1.2%, 17.1% and 23.5%, respectively, compared to control cells. Control RIN-5F cells cultured in high glucose medium showed gluco-toxicity and it showed 40% reduction in cell proliferation than the control cells in normo-glyceamic medium. (CA+24-MCA) treated cells (0.1 μg/ml, 1 μg/ml, 10 μg/ml doses) in high glucose medium showed significant protection from gluco-toxicity dose dependently and the percentage(s) of cell growth were 23.6%, 92.5% and 115% respectively to that of control cells in high glucose medium. The cell proliferation in 0.1% DMSO, 0.1 μg/ml, 1 μg/ml and 10 μg/ml doses of (CA+24-MCA) in high glucose medium was compared with the cell proliferation in same drug concentration in 5.5 mM glucose media (pair wise). Control RIN-5F cells and 0.1 μg/ml (CA+24-MCA) treated cells in high glucose medium showed reduction in cell growth of 40 and 25.8%, respectively, than control cells in normal glucose medium. In higher doses (1 μg/ml and 10 μg/ml) of (CA+24-MCA)-treated cells no significant difference in cell growth was observed between higher and normal glucose medium in respective doses (Fig 7).

### Cytoprotective effect of (CA+24-MCA) from glucotoxicity in RIN-5F cell by fluorescent microscopy

**Fig 8:**
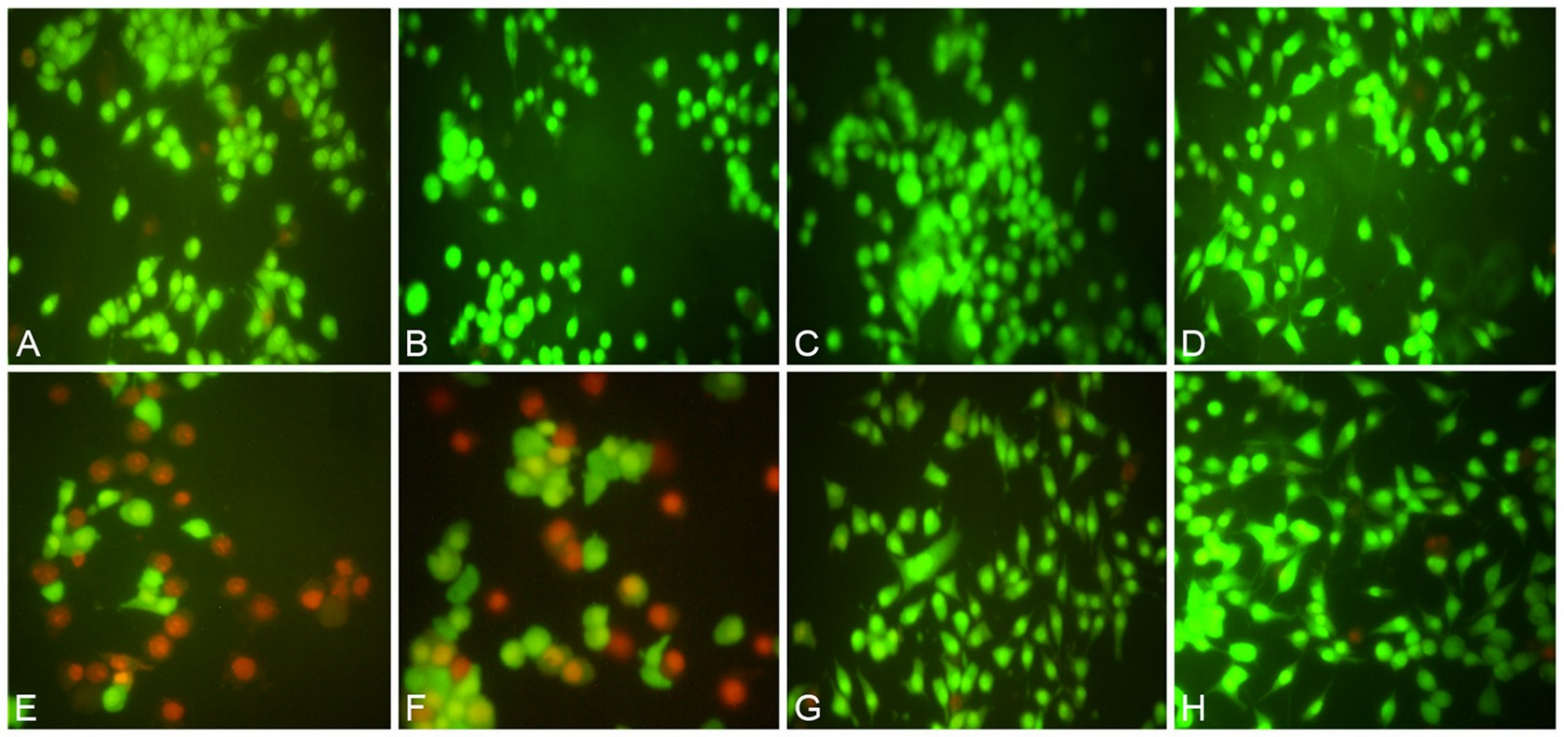
A—Control RIN-5F cells cultured in medium containing 5.5 mM glucose treated with 1% DMSO; B—RIN-5Fcells cultured in medium containing 5.5 mM glucose treated with 0.1 μg/ml (CA+24-MCA); C—RIN-5F cells cultured in medium containing 5.5 mM glucose treated with 1 μg/ml (CA+24-MCA); D—RIN-5F cells cultured in medium containing 5.5 mM glucose treated with 10 μg/ml (CA+24-MCA); E—Control RIN-5F cells cultured in medium containing 25 mM glucose treated with 1% DMSO; F—RIN-5F cells cultured in medium containing 25 mM glucose treated with 0.1 μg/ml (CA+24-MCA); G—RIN-5F cells cultured in medium containing 25 mM glucose treated with 1 μg/ml (CA+24-MCA); H—RIN-5F cells cultured in medium containing 25 mM glucose treated with 10 μg/ml (CA+24-MCA). Magnification of cells: 400X; Live and dead cells appeared in green and red colours, respectively.

In fluorescent microscopy of RIN-5F cells cultured in normal glucose (5.5 mM) medium treated with 0.1% DMSO, 0.1 μg/ml, 1 μg/ml or 10 μg/ml of (CA+24-MCA) for 48 h, the control (0.1% DMSO treated) cells didn’t show any significant loss of cell viability and only limited cell death was observed (Fig 8A). (CA+24-MCA) (0.1 μg/ml, 1 μg/ml or 10 μg/ml)-treated cells didn’t show any cytotoxicity but increase in cell proliferation was observed dose dependently (all cells appeared in green colour) (Fig 8B–8D). RIN-5F cells when cultured in high glucose (25 mM) medium and treated with 0.1% DMSO, 0.1 μg/ml, 1 μg/ml or 10 μg/ml of (CA+24-MCA) for 48 h, the control cells (treated with 0.1% DMSO) showed significant cell death due to the high glucose induced toxicity; majority of cells were dead and appeared in red colour and limited number of cells were viable (green colour, Fig 8E). (CA+24-MCA) 0.1 μg/ml, 1 μg/ml or 10 μg/ml-treated cells in high glucose medium showed a concentration-dependent cytoprotection in RIN-5F cells from high glucose toxicity. (CA+24-MCA) 0.1 μg/ml-treated cells showed increment in number of viable cells (green colour) with limited dead cells (red colour, Fig 8F). (CA+24-MCA) 1 μg/ml or 10 μg/ml-treated cells showed significant increase in cytoprotection from high glucose toxicity (Fig 8G and 8H), and significant increase in cell viability was also observed. In comparison, the morphology of cells in panels G and H vary when compared with E (control) and F (0.1 μg/ml, CA+24-MCA). In (E and F), cells (mostly dead cells) were enlarged (and round) due to high glucose induced toxicity and absence of cytoplasmic processes. In 1 μg/ml and 10 μg/ml CA+24-MCA-treated cells in high glucose medium (G, H), cells were protected from glucose toxicity and they retained their normal morphology as in normal glucose medium. Cells which appeared in green colour were comparable to the cell viability of RIN-5F cells cultured in normoglyceamic medium at the same concentrations.

### Insulin release from RIN-5F cells by (CA+24-MCA)

**Fig 9:**
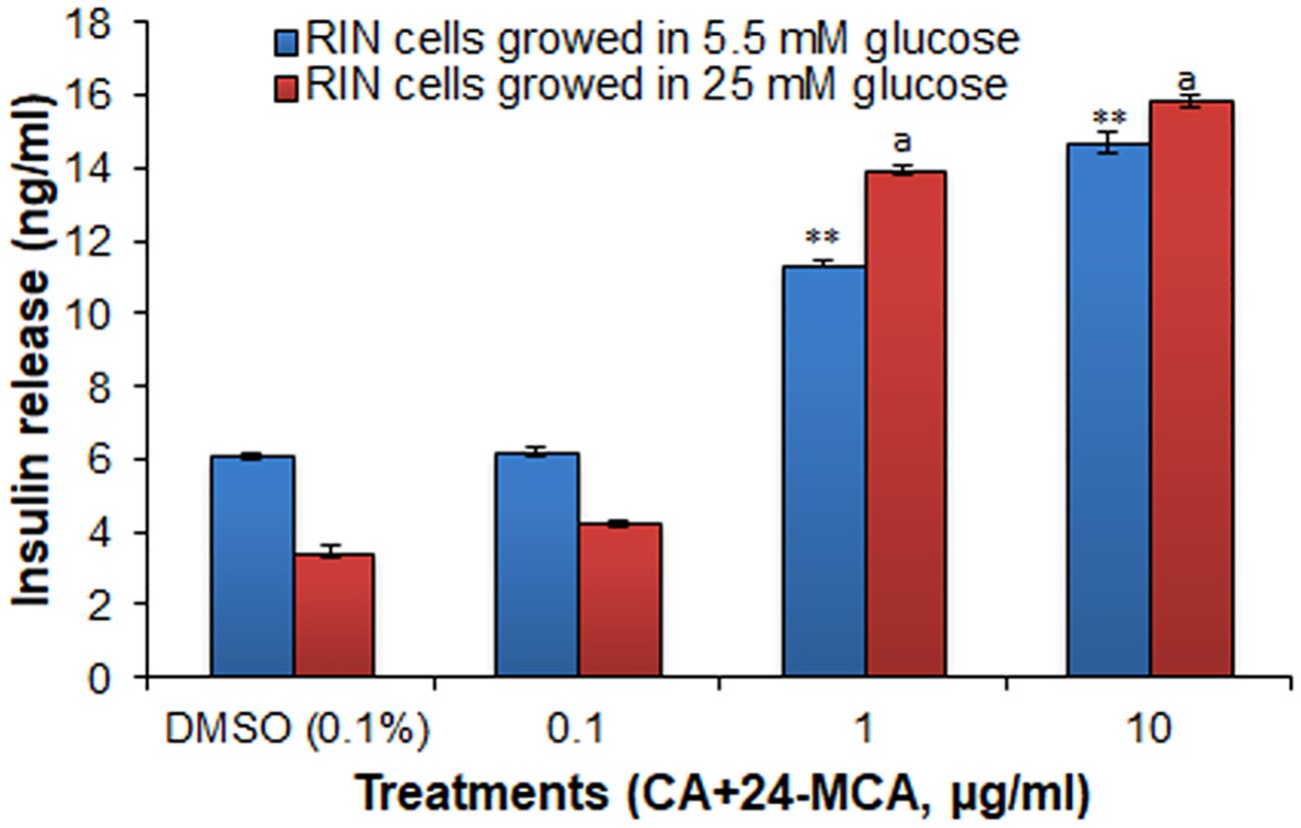
Effect of (CA+24-MCA) isolated from hexane extract of *F*. *krishnae* on insulin release from RIN-5F cells cultured in normal/high glucose medium. (CA+24-MCA), cycloartenol (CA) + 24-methylenecycloartanol (24-MCA); values are mean ± S.D.; n = 3; ** P < 0.001 (compared to control cells cultured in 5.5 mM glucose); ^a^P < 0.001 (compared to control cells cultured in 25 mM glucose).

Insulin release assay on RIN-5F cells cultured in normoglyceamic medium with the 0.1 μg/ml, 1 μg/ml or 10 μg/ml of (CA+24-MCA) showed significant increase in insulin release dose dependently (Fig 9), and the percentage increase(s) in insulin release were 1.1%, 88.5% and 140.9%, respectively, compared to the control cells. Insulin release from RIN-5F cells cultured in high glucose medium with 0.1 μg/ml, 1 μg/ml or 10 μg/ml of (CA+24-MCA) also showed significant increase in insulin release dose dependently. The percentage increases in insulin release were 23.1%, 304.3% and 359.4%, respectively, to the control cells (Fig 9).

### Subacute toxicity evaluation of (CA+24-MCA) in mice

In short term toxicity evaluation, 15, 75 and 150 mg/kg of (CA+24-MCA) didn’t show any conspicuous toxicity in mice (Table 4). Serum ALP, creatinine, protein and triglyceride values were unaltered even at the highest dose tested (150 mg/kg) compared to those of control animals. Serum glucose and cholesterol values showed a significant reduction in all three doses tested (15, 75 and 150 mg/kg), while serum GPT, GOT and urea values showed a significant reduction only in 75 and 150 mg/kg body weight. Hemoglobin content, peritoneal macrophages, WBC count, food and water intake and body weight in all three doses of (CA+24-MCA) didn’t show any significant deviation from the control values. Control and test animals exhibited no behavioral changes and their internal organs were devoid of any significant pathological and morphological changes.

**Table 4:** Effect of (CA+24-MCA) isolated from hexane extract of *F*. *krishnae* on serum biochemical parameters and hemoglobin, white blood cells and peritoneal macrophages in subacute (30 days) toxicity study in mice.

Groups	Control	15 mg/kg	75 mg/kg	150 mg/kg
SGPT (IU/l)	49.0 ± 4.8	48.6 ± 3.2	37.0 ± 3.0	36.0 ± 3.3
SGOT (IU/l)	112.0 ± 5.1	110.2 ± 3.6	90.0 ± 2.1*	91.0 ± 2.2*
ALP (KAU)	9.5 ± 0.27	9.3 ± 0.35	9.0 ± 0.50	8.80 ± 0.32
Urea (mg/dl)	42.4 ± 3.4	32.1 ± 2.1	30.3 ± 1.7*	30.2 ± 2.5*
Cholesterol (mg/dl)	175.2 ± 8.7	148.4 ± 5.0**	155.2 ± 6.1*	153.1 ± 5.2*
Glucose (mg/dl)	74.5 ± 4.2	60.1 ± 2.1*	59.2 ± 3.9*	60.3 ± 1.8*
Creatinine (mg/dl)	1.5 ± 0.18	1.4 ± 0.20	1.5 ± 0.16	1.5 ± 0.21
Protein (mg/dl)	6.4 ± 0.71	6.7 ± 0.91	5.9 ± 0.21	6.3 ± 0.65
Triglyceride (mg/dl)	129.1 ± 6.8	128.3 ± 7.6	139.0 ± 5.0	123.1 ± 6.2
Hb (mg %)	13.0 ± 0.63	13.3 ± 0.47	13.3 ± 0.47	13.5 ± 0.72
WBC (10^3^/μl)	15.0 ± 0.80	15.4 ± 0.54	15.3 ± 0.58	15.3 ± 0.38
Macrophages (10^6^/mouse)	8.0 ± 0.38	7.9 ± 0.35	8.1 ± 0.34	7.8 ± 0.78
Body weight (g)	36.6 ± 1.21	36.1 ± 1.54	36.0 ± 2.75	36.6 ± 2.82

SGPT, Serum glutamate pyruvate transaminase; SGOT, Serum glutamate oxalate transaminase; ALP, Alkaline phosphatase. Values are mean ± S.D.; n = 6;

* P < 0.05;

** P < 0.01 (compared to control).

## Discussion

*F*. *krishnae*, though traditionally known, is least studied at molecular level for its antidiabetic activity, and thus we followed activity guided isolation to obtain its bioactive molecule(s) and pursued their mechanism of action. FKEH, FKEHF4 and (CA+24-MCA) showed significant glucose lowering activity in OGTT. Both CA and 24-MCA belong to the group of 4,4-dimethyl phytosterols (triterpene alcohols), with two methyl groups at C4 of the aliphatic A-ring. In several studies, these two triterpene alcohols were isolated together due to their more or less similar structural features (with only minor differences at the tail ends of their side chain carbons) [27–29]. CA and 24-MCA were also found together in many vegetable oils [30, 31]. One example is, CA and 24-MCA are the major triterpene alcohols in rice bran oils [32]. Moreover, CA is a major precursor molecule for the biosynthesis of various phytosterols [33–36].

*Ficus* species are known for antidiabetes activity in *in vivo* models and their known bioactive metabolites are triterpenoids, sterols, flavonoids, phenolic acids, alkaloids, glycosides, coumarins, tannins and vitamin E [10]. Further, many triterpenoids of plant origin are reported to have antidiabetes activity, and they are known to ameliorate diabetic complications like retinopathy, neuropathy, myopathy, nephropathy and impaired wound healing. Some examples of triterpenoids with such activities are corosolic acid, rhododendric acid, moronic acid, morolic acid, lupenone, hopane-6α-22-diol, karaviloside XI, momordicoside S, momordicine II, kuguaglycoside G, pachymic acid, ginsenoside, ursolic acid, astragaloside IV, asiatic acid and maslinic acid [37, 38]. On this background, we further investigated the initial leads obtained for the mixture of two terpenoid alcohols (CA+24-MCA) isolated from *F*. *krishnae*.

In HFD-STZ diabetes rats, (CA+24-MCA) significantly corrected the deranged blood glucose values. Liver weight, liver glycogen content and serum biochemical parameters are significant in the progression and pathogenesis of diabetes, and here in drug (CA+24-MCA)-treated diabetic animals, these deranged values were normalized. In case of high fat diet control animals, serum insulin level appeared to be high, and this is because beta cells were signaled to release higher concentration of insulin necessary to meet the metabolic requirements initiated by the high fat diet. Even though the insulin level was higher on 15th day in HFD-STZ diabetic control animals, the blood glucose level was not lowered, and this is due to the insulin resistance associated with type II diabetes. Due to the partial destruction of pancreatic beta cell mass and deranged glucose metabolism, type II diabetes progresses from insulin resistance to insulin insufficiency [17, 39, 40]. In this study, (CA+24-MCA) ameliorated the altered insulin resistance and normalized serum insulin level in diabetic animals. It may be due to the cumulative effect of the drug in pancreatic beta cell protection from glucotoxicity and enhancement in the growth and differentiation of beta cells population. Besides, the drug (CA+24-MCA)-treatment normalized serum GOT, GPT, urea, triglycerides, total cholesterol, creatinin, albumin and protein values of diabetic animals to normal levels. This correction was either through proper insulin release by the drug and normalization of glucose metabolism and/or through the inhibition of gluconeogenesis from liver glycogen and muscle proteins.

Insulin resistance seen associated with type II diabetic subjects influences the up regulation of cholesterol biosynthesis. CA is known to have hypolipedimic activity in diabetic rats [31]. Another target of phytosterols in lipid homeostasis is apolipoprotein (apo A-I), which is the major protein involved in the transportation and metabolism of lipids and is an integral component in high-density lipoprotein (HDL). CA along with β-sitosterol is known to effectively correct the decrease in apo A-1 levels in cholesterol fed Wistar rats (compared to the treatment of β-sitosterol only) [41]. In another study in high fat diet fed C57BL/6J mice, triterpene alcohol and sterol preparation (TASP) from rice bran lowered the diet induced glucose-dependent insulinotropic peptide, up regulated the gene expression of fatty acid oxidation-related gene and down regulated the fatty acid synthesis related gene. Daisuke and co-workers showed that CA and 24-MCA cause less body weight gain, promote fat utilization, enhance the expression of genes associated with lipid oxidation and suppress the gene expression related to lipid biosynthesis. Besides, CA and 24-MCA specifically lowered the gene expression of sterol regulatory element-binding protein (SREBP)-1c. By these actions, the triterpene alcohols and sterols prevented diet induced obesity [42, 43]. Shimotoyodome and co-workers recently showed that the consumption of brown rice, with enriched contents of CA and 24-MCA, led to a lower postprandial hyperglycemia [44, 45]. Further, Tanaka and co-workers demonstrated the antihyperglyceamic effects of cycloartanol and 24-MCA along with lophenol, 24-methyl-lophenol, 24-ethyl-lophenol isolated from *Aloe vera* gel in type II diabetic BKS.Cg-m(+/+)Lepr(db/J) (db/db) mice [46]. The secondary starvation which existed in diabetic scenario enhanced protein degradation and deranged oxidative deamination of amino acids leading to the formation of excess ammonia culminating in the formation of higher levels of serum urea. In later stages, the consistently elevated urea level in blood leads to the dysfunctioning of kidneys, resulting in deranged albumin and creatinin levels. These conditions were abolished by the drug (CA+24-MCA) in diabetic animals, which was reflected in the normalization of the serum urea, albumin, protein and creatinin values. In addition to the antidiabetes activity, CA possessed a broad range of pharmacological activities such as anti-inflammatory, antitumor, antioxidant, antibiosis and anti-Alzheimer’s disease [47].

In our study, in normoglyceamic medium, 0.1, 1.0 and 10 μg/ml doses of (CA+24-MCA) showed concentration dependent increment in RIN-5F cell proliferation (in MTT assay). Previous studies showed glucotoxicity, due to high levels of glucose in blood serum, as a major cause in the pathogenesis of diabetes [48]. RIN-5F control cells in high glucose medium showed low insulin secretion. One of the reasons for this reduction in insulin secretion of control RIN-5F cells in high glucose medium is the lower number of viable cells due to glucotoxicity. At the same time, the inherent capacity of remaining cells in control group to release basal insulin level in high glucose condition is the reason for normalization of insulin release, but it is still less than the insulin release from RIN-5F cell in normal glucose medium. In RIN-5F cells grown in high glucose medium, the drug showed dose dependent protection from glucotoxicity and significant increment in cell growth. These results are in correlation with the data obtained from the fluorescent microscopic images. In normal and high glucose medium, the enhanced secretion of insulin by RIN-5F cells compared to control cells is due to the insulin secretagogue activity of (CA+24-MCA). In a nutshell, the increment in insulin release is due to the cumulative effect of the drug on cytoprotection of RIN-5F cells from glucotoxicity, beta cell proliferation and insulin secretagogue activity of (CA+24-MCA). Cytoprotection and beta cell proliferation are important targets for the treatment of type II diabetes. Plant derived compounds (including terpenoids) are reported to show beta cell protection from hyperglycemia and amelioration of diabetic complications [38, 49–52].

In short term toxicity evaluation in mice, (CA+24-MCA) didn’t show any conspicuous toxic symptoms even at 150 times higher concentration of the therapeutic dose. Body weight, food and water intake and behavioral activities of the animals were normal. Serum hematological and biochemical parameters were normal and the drug at 15, 75 and 150 mg/kg showed a significant reduction in glucose, cholesterol, urea and SGOT values. Though the drug (CA+24-MCA) has glucose lowering action, it didn’t exert any hypoglycemic shock even at higher doses and maintained the glucose level close to normal control values. The reduction in serum cholesterol, urea and GOT values are additive qualities of the drug, and due to these effects diabetic complications such as hypercholestremia, nephropathy, cardiovascular issues and liver damage can be ameliorated by the drug. Briefly, in this study, we validated the antidiabetes activity of *F*. *krishnae* and isolated its antidiabetes compounds, (CA+24-MCA).

## Conclusions

*F*. *krishnae* is a new source of the two phytosterols (CA+24-MCA), which exert antidiabetes activity by protecting beta cells from glucotoxicity associated with diabetes, enhancing beta cell population and normalizing insulin release from pancreatic beta cells. These terpenoids (CA+24-MCA) also have the potency to ameliorate the altered serum biochemical parameters in diabetic animals to normal level. In short term toxicity evaluation (CA+24-MCA) is devoid of any conspicuous toxic symptoms even at 150 times higher dose of the therapeutic dose, which is an additive quality of the drug. So, (CA+24-MCA) is a promising candidate for the development of new generation antidiabetes drugs.

## Supporting information

S1 Scheme Isolation of (CA+24-MCA) from the stem bark of *Ficus krishnae*. (DOCX)

S1 Table 1H NMR (CDCl_3_, 400 MHz, δ ppm) and 13C NMR (CDCl_3_, 100 MHz, δ ppm) assignments of cycloartenol (CA) and 24-methylenecycloartanol (24-MCA). (DOCX)

S1 Fig 1H NMR spectrum of (CA+24-MCA). (DOCX)

S2 Fig 13C NMR spectrum of (CA+24-MCA). (DOCX)

S3 Fig Mass spectrum of (CA+24-MCA). (DOCX)

S4 Fig Gas chromatogram of chemically converted (CA+24-MCA), displaying the two derivatized peaks of CA and 24-MCA. (DOCX)
