# Supplementary material for: Antidiabetes constituents, cycloartenol and 24-methylenecycloartanol, from Ficus krishnae
Source: PLoS One. 2020 Jun 25;15(6):e0235221. doi: 10.1371/journal.pone.0235221 (PMC7316276; doi:10.1371/journal.pone.0235221)
Supplement: S1 Scheme — (DOCX) [file pone.0235221.s001.docx]

Scheme S1. Isolation of (CA+24-MCA) from the stem bark of *Ficus krishnae*.

*Ficus krishnae* stem bark

(Dry powder, 500 g)

Water extract (24.0 g)

Methanol, Soxhlet extraction (3 L, 24 h)

Hexane, Soxhlet extraction (3 L, 24 h)

1H NMR, 13C NMR, Mass spectrometry

FKEHF4 (2.0 g), Silica gel 100-200 column chromatography

FKEHF2

(5.42 g)

(Cycloartenol (CA) + 24-methlenecycloartanol (24-MCA))

White crystalline solid (100 mg, 0.0031% w/w, dr. wt.)

Major compound (1.60 g)

Minor compound (0.40 g)

FKEHF7

(0.09 g)

FKEHF6

(0.36 g)

FKEHF3

(2.04 g)

FKEHF1

(0.17 g)

FKEHF5

(0.79 g)

FKEHF4

(2.83 g)

Silica gel 60-120 column chromatography

Methanol extract (59.0 g)

Hexane extract (12.0 g)

Water, Cold extraction (2 L, 24 h)

Sequential extraction
