# Supplementary material for: Antidiabetes constituents, cycloartenol and 24-methylenecycloartanol, from Ficus krishnae
Source: PLoS One. 2020 Jun 25;15(6):e0235221. doi: 10.1371/journal.pone.0235221 (PMC7316276; doi:10.1371/journal.pone.0235221)
Supplement: S1 Table — (DOCX) [file pone.0235221.s002.docx]

Table S1. 1H NMR (CDCl_3_, 400 MHz, δ ppm) and 13C NMR (CDCl_3_, 100 MHz, δ ppm) assignments of cycloartenol (CA) and 24-methylenecycloartanol (24-MCA).

| Carbon number | Cycloartenol (CA) | | 24-Methylenecycloartanol (24-MCA) | |
| --- | --- | --- | --- | --- |
|  | 13C NMR (100 MHz) δ ppm | 1H NMR  (400 MHz) δ ppm | 13C NMR (100 MHz) δ ppm | 1H NMR (400 MHz) δ ppm |
| C-1 | 32.90 | - | 32.90 | - |
| C-2 | 31.32 | - | 31.32 | - |
| C-3 | 78.86 | 3.29 (1H, m) | 78.86 | 3.29 (1H, m) |
| C-4 | 40.49 | - | 40.49 | - |
| C-5 | 47.71 | - | 47.71 | - |
| C-6 | 21.89 | - | 21.89 | - |
| C-7 | 26.03 | - | 26.03 | - |
| C-8 | 48.01 | - | 48.01 | - |
| C-9 | 20.00 | - | 20.00 | - |
| C-10 | 26.48 | - | 26.48 | - |
| C-11 | 26.48 | - | 26.48 | - |
| C-12 | 33.81 | - | 33.81 | - |
| C-13 | 45.30 | - | 45.30 | - |
| C-14 | 48.81 | - | 48.81 | - |
| C-15 | 35.89 | - | 35.89 | - |
| C-16 | 28.17 | - | 28.17 | - |
| C-17 | 52.29 | - | 52.29 | - |
| C-18 | 18.05 | 0.81 (3H, s, CH_3_) | 18.05 | 0.81 (3H, s, CH_3_) |
| C-19 | 29.92 | 0.33 (1H, d, 3.2 Hz, Ha)  0.55 (1H, d, 3.2 Hz, Hb) | 29.92 | 0.33 (1H, d, 3.2 Hz, Ha)  0.55 (1H, d, 3.2 Hz, Hb) |
| C-20 | 36.13 | - | 36.13 | - |
| C-21 | 18.31 | 0.89 (3H, d, J = 6.2 Hz, CH_3_) | 18.31 | 0.89 (3H, d, J = 6.2 Hz, CH_3_) |
| C-22 | 36.36 | - | 35.58 | - |
| C-23 | 25.42 | - | 31.97 | - |
| C-24 | 125.27 | 5.10 (1H, t, J = 5.6 Hz) | 156.91 | - |
| C-25 | 130.69 | - | 34.98 | 2.24 (1H, sep) |
| C-26 | 17.67 | 1.61 (3H, s, CH_3_) | 21.14 | 1.04 (3H, d, J = 2.3 Hz, CH_3_) |
| C-27 | 25.75 | 1.69 (3H, s, CH_3_) | 22.02 | 1.02 (3H, d, J = 2.3Hz, CH_3_) |
| C-28 | 26.03 | 0.97 (3H, s, CH_3_) | 24.95 | 0.97 (3H, s, CH_3_) |
| C-29 | 14.0 | 0.88 (3H, s, CH_3_) | 14.0 | 0.89 (3H, s, CH_3_) |
| C-30 | 19.64 | 0.87 (3H, s, CH_3_) | 19.64 | 0.87 (3H, s, CH_3_) |
| C-31 | - | - | 105.93 | 4.67 (1H, brs, H-a)  4.71 (1H, d, J = 1Hz, H-b) |
