# Supplementary figures and images for: Antidiabetes constituents, cycloartenol and 24-methylenecycloartanol, from Ficus krishnae
Source: PLoS One. 2020 Jun 25;15(6):e0235221. doi: 10.1371/journal.pone.0235221 (PMC7316276; doi:10.1371/journal.pone.0235221)

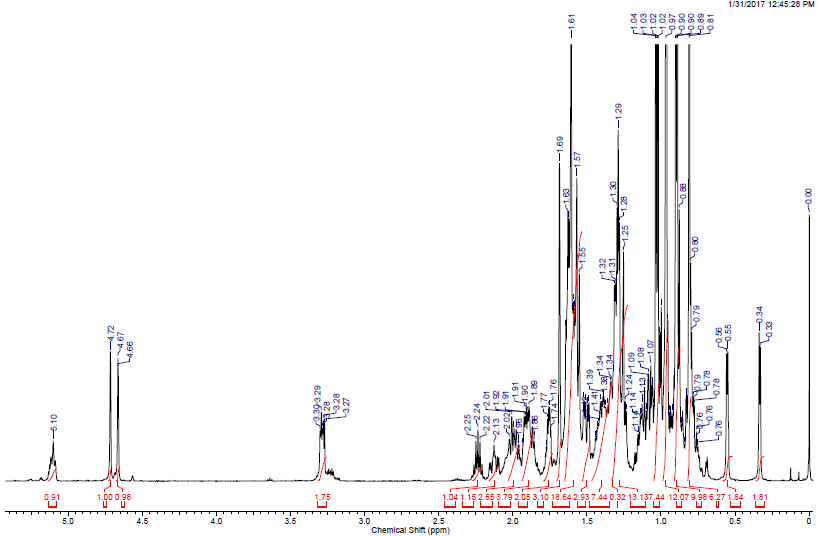


Fig. S1. 1H NMR spectrum of (CA+24-MCA).

Supplement: S1 Fig — (DOCX) [file pone.0235221.s003.docx]
