# Supplementary material for: Antidiabetes constituents, cycloartenol and 24-methylenecycloartanol, from Ficus krishnae
Source: PLoS One. 2020 Jun 25;15(6):e0235221. doi: 10.1371/journal.pone.0235221 (PMC7316276; doi:10.1371/journal.pone.0235221)
Supplement: S2 Fig — (DOCX) [file pone.0235221.s004.docx]

Fig. S2. 13C NMR spectrum of (CA+24-MCA).
